# Deep Learning–Based Classification of Melanoma Using Dermoscopic Images: Comparative Evaluation of Convolutional Neural Network and DenseNet Models

**DOI:** 10.2196/104086

**Published:** 2026-10-27

**Authors:** Harshal Sanghvi, Argyrios Agritis, Isabella Alvarez, Mitsu Patel, Shuwen Wang, Riki Patel, Raphael Banoub, Timonthy Makkar

**Affiliations:** 1 Florida Atlantic University, 777 Glades Road, Boca Raton, FL, 33431, United States, 1 5615634452; 2 Department of Technology and Clinical Trials, Advanced Research LLC, Pompano Beach, FL, United States; 3 Clemson University, Clemson, SC, United States; 4 Mayo Clinic in Florida, Jacksonville, FL, United States; 5 Department of Dermatology, Rutgers New Jersey Medical School, Brunswick, NJ, United States

**Keywords:** deep learning, dermoscopy, medical image analysis, data augmentation, melanoma detection

## Abstract

**Background:**

For melanoma, an aggressive form of skin cancer, early and accurate detection is critical to improving clinical outcomes. Deep learning–based image analysis has emerged as a promising approach for computer-assisted evaluation of dermoscopic lesions. However, model performance is often affected by limited dataset size, class imbalance, overfitting, and differences in network architecture and optimization strategies.

**Objectives:**

This study was aimed at developing and comparatively evaluating convolutional neural network (CNN) and DenseNet-based deep learning models for automated classification of dermoscopic skin lesion images. The effects of data augmentation, network architecture, optimizer selection, and training duration on classification performance were examined.

**Methods:**

Publicly available dermoscopic skin lesion images obtained from Kaggle were evaluated using a conventional CNN, DenseNet169, and DenseNet201. The original dataset contained 2357 images representing multiple benign and malignant skin lesion categories. Images were resized to 250×250 pixels, labeled, and randomly partitioned into training and testing sets at an 80:20 ratio. To address class imbalance, image augmentation using transformations, including ±10° shear, was performed, increasing the dataset size to 6857 images, with approximately 500 additional samples generated per class. Models were trained using adaptive moment estimation (Adam) and root mean square propagation (RMSprop) optimizers, and evaluated after 20, 60, and 100 epochs. Performance was assessed using accuracy, precision, recall (sensitivity), specificity, *F*_1_-score, confusion matrices, and training and validation loss.

**Results:**

Data augmentation substantially improved model performance and reduced evidence of overfitting compared with the original dataset. On the augmented dataset, DenseNet169 and DenseNet201 achieved accuracy and *F*_1_-scores exceeding 94% after 100 epochs, consistently outperforming the conventional CNN. DenseNet169 achieved up to 95% validation accuracy, with only minor performance differences between the Adam and RMSprop optimizers. The CNN showed greater susceptibility to overfitting on the smaller dataset; its *F*_1_-score, calculated over the complete dataset, increased from 31% after 20 epochs to 59% after 60 epochs and 87% after 100 epochs before augmentation. Across the comparative experiments, the DenseNet architectures demonstrated more stable validation performance and better generalization following augmentation.

**Conclusions:**

DenseNet169 and DenseNet201 demonstrated stronger and more stable classification performance than the conventional CNN for dermoscopic skin lesion classification, particularly after augmentation of the training dataset. Increasing the dataset size and improving class balance led to reduced overfitting and improved model performance. These findings support further investigation of DenseNet-based approaches as potential clinical decision support tools for melanoma and skin lesion assessment. However, prospective evaluation using independent, multicenter datasets and diverse patient populations is required before clinical implementation.

## Introduction

Skin cancer is one of the most prevalent malignancies globally, accounting for approximately 40%‐50% of all cancer diagnoses within the United States. It is broadly categorized into melanoma and nonmelanoma skin cancers (NMSCs) [1]. NMSCs primarily originate from keratinized epithelial cells and include basal cell carcinoma, squamous cell carcinoma, and sebaceous gland carcinoma. Although melanoma is less common, it represents the most aggressive form of skin cancer and accounts for the majority of skin cancer–related deaths [2].

Melanoma arises from melanocytes, which are pigment-producing cells localized in the basal layer of the epidermis. Exposure to UV radiation induces keratinocytes to secrete α-melanocyte-stimulating hormone, which activates melanocytes via the melanocortin 1 receptor, thereby promoting melanin synthesis [3]. This physiological response confers photoprotective properties; however, chronic UV exposure can promote DNA damage and mutagenesis, contributing to malignant transformation and unregulated cellular proliferation. Epidemiological data reveal sex- and age-specific variations in melanoma incidence: incidence is higher in females than in males before the age of 40 years, whereas by the age of 75 years, incidence in males is nearly 3 times that in females. Both genetic predisposition and environmental factors contribute to melanoma pathogenesis, with UV radiation implicated in approximately 60%‐70% of cases. The World Health Organization classifies UVA (315‐400 nm) and UVB (280‐315 nm) radiation as complete carcinogens, given their involvement in tumor initiation and progression [4].

Clinically, melanoma is classified into distinct histopathological variants, of which superficial spreading melanoma is the most common, especially in populations with lighter skin phototypes [5]. Despite advancements in understanding melanoma biology, its global incidence continues to rise, emphasizing the importance of early detection in improving patient prognosis. Diagnostic assessment relies predominantly on dermoscopic evaluation, supplemented by heuristic approaches such as the ABCDE (asymmetry, border irregularity, color heterogeneity, diameter, and evolution) criteria. Nevertheless, these methods are inherently subjective, depend on clinical expertise, and are susceptible to interobserver variability [6].

To address these diagnostic challenges, AI and computer-aided diagnosis systems have been developed for automated skin lesion analysis. Deep learning frameworks, particularly convolutional neural networks (CNNs), have demonstrated diagnostic performance comparable to that of experienced dermatologists [7]. Model performance has been improved by incorporating transfer learning and sophisticated feature extraction techniques. Public datasets, such as those from the International Skin Imaging Collaboration and the PH^2^ database, have been instrumental in training and validating AI models [8].

Complementary to algorithmic innovations, the use of image preprocessing techniques, such as top-hat filtering and DullRazor for hair artifact removal and noise reduction, has substantially improved lesion segmentation and classification accuracy [9]. Clinical technologies, including digital dermoscopy, total body photography, and mobile health platforms, have enhanced the accessibility of skin surveillance. However, challenges remain regarding cost, patient adherence to follow-up, and variability in diagnostic precision [10].

In this study, we propose a novel deep learning framework that integrates transfer learning with densely connected neural network (DenseNet) architectures (DenseNet169 and DenseNet201) and a conventional CNN model for automated skin lesion classification. The framework iteratively optimizes hyperparameters to enhance diagnostic accuracy and is benchmarked against established machine learning counterparts. By combining rigorous preprocessing protocols with state-of-the-art neural network architectures, this work aims to promote early melanoma detection and support clinical decision-making.

Table 1 summarizes the performance metrics of the proposed approach and relevant methods reported in the literature.

**Table 1.** Performance overview of the proposed method and relevant methods reported in the literature.

Reference	Methods	Dataset/validation	Key findings	Sensitivity/accuracy
This study	DenseNet169, DenseNet201, and CNN^a^	Dermoscopic images	Validation and training accuracy greater than 98%	Accuracy 98%
Cheong et al [11], 2021	Feature extraction (entropy, LBP^b^, GLCM^c^, and ABCDE^d^ criteria) with SVM-RBF^e^	Dermoscopic images	Classification accuracy greater than 97.5%	Sensitivity greater than 97.5%
Waheed et al [12], 2023	Deep learning (CNN, hybrid CNN, and transfer learning) with data augmentation	ISIC^f^ dataset	Correct classification rate of 81%	Sensitivity 92.46%; specificity 92.23%
Alsaade et al [13], 2021	Hybrid ML^g^ and DL^h^ (segmentation, CNN, and ANN^i^)	ISIC 2018 and PH^2^^j^	ANN accuracy of 98% (ISIC) and 97.5% (PH^2^)	Sensitivity 83%‐97.5%
Indraswari et al [14], 2022	Transfer learning with MobileNetV2	ISIC Archive	Lightweight model suitable for mobile deployment	Accuracy 85%; sensitivity 85%
Tumpa and Kabir [15], 2021	Image preprocessing (MGI^k^ for hair removal), texture and shape features, and ANN	ISIC and PH^2^	Benign versus malignant lesions classified with 97.7% accuracy	Sensitivity ~97%
Brinker et al [16], 2019	CNN compared with dermatologists	ISIC Archive	CNN sensitivity comparable to that of dermatologists; higher specificity (68.2% vs 64.4%)	Sensitivity 89.4%
Thaajwer and Ishanka [17], 2020	Image processing (GLCM and color/shape features)+SVM^l^	Dermoscopic images	Classification accuracy of 83%	Not reported
Banerjee et al [18], 2020	Deep learning, fuzzy logic, and DCNN^m^ (YOLO^n^)	PH^2^, ISBI 2017, ISIC 2019	High accuracy across datasets	Sensitivity 97.5%‐98.5%
Bakheet and El-Nagar [19], 2021	Real-time computer-aided diagnosis with DNN^o^	PH^2^ dataset	Accuracy of ~97.5%	Sensitivity 100%
Chang et al [20], 2022	CNN, XGB^p^ classifier, and SMOTE^q^-based balancing	Stratified 10-fold cross-validation	Accuracy of ~97.5%	Sensitivity 98.1%
Thiyaneswaran et al [21], 2021	GLCM features with ML classifiers	Dermoscopic images	Accuracy of ~90%	Positive prediction 95%; negative prediction 80%

a CNN: convolutional neural network.

b LBP: local binary pattern.

c GLCM: gray level co-occurrence matrix.

d ABCDE: asymmetry, border irregularity, color heterogeneity, diameter, and evolution.

e SVM-RBF: support vector machine with the radial basis function kernel.

f ISIC: International Skin Imaging Collaboration.

g ML: machine learning.

h DL: deep learning.

i ANN: artificial neural network.

j PH2: PH2 Dermoscopic Image Database.

k MGI: morphological gradient image.

l SVM: support vector machine.

m DCNN: deep convolutional neural network.

n YOLO: you only look once.

o DNN: deep neural network.

p XGB: extreme gradient boosting.

q SMOTE: synthetic minority oversampling technique.

## Methods

### Overview

Images of skin lesions were used as inputs for the CNN, DenseNet169, and DenseNet201 models. The images were read using the OpenCV (cv2) library at a size of 250×250 pixels to speed up processing and reduce memory consumption. Subsequently, the images were labeled, and a label binarizer was used to convert the multiclass labels to binary labels. The dataset was randomly partitioned into training (80%) and validation (20%) sets. Each model was trained for 20, 60, and 100 epochs. The CNN model incorporated 3 Conv2D layers with a kernel size of 3×3 and stride of 2×2. The adaptive moment estimation (Adam) and root mean square propagation (RMSprop) optimizers were used to optimize training conditions for the models. Accuracy and loss over the training and validation data were recorded. Loss was calculated as the difference in the actual and predicted class values.

### Dataset

The dataset used for classification was obtained from Kaggle. The classification models included a CNN and 2 DenseNet architectures (DenseNet169 and DenseNet201). The dataset included images depicting several forms of skin lesions: actinic keratosis, basal cell carcinoma, dermatofibroma, melanoma, nevus, pigmented benign keratosis, seborrheic keratosis, squamous cell carcinoma, and vascular lesions.

We evaluated dataset versions 1 and 2. Both datasets contained images illustrating the same conditions. However, dataset 2 included additional images in each category and exhibited a more balanced distribution of data across conditions. Dataset 2 was used after dataset 1 to help mitigate model overfitting. Several images from both datasets are shown in Figure 1.

**Figure 1.**
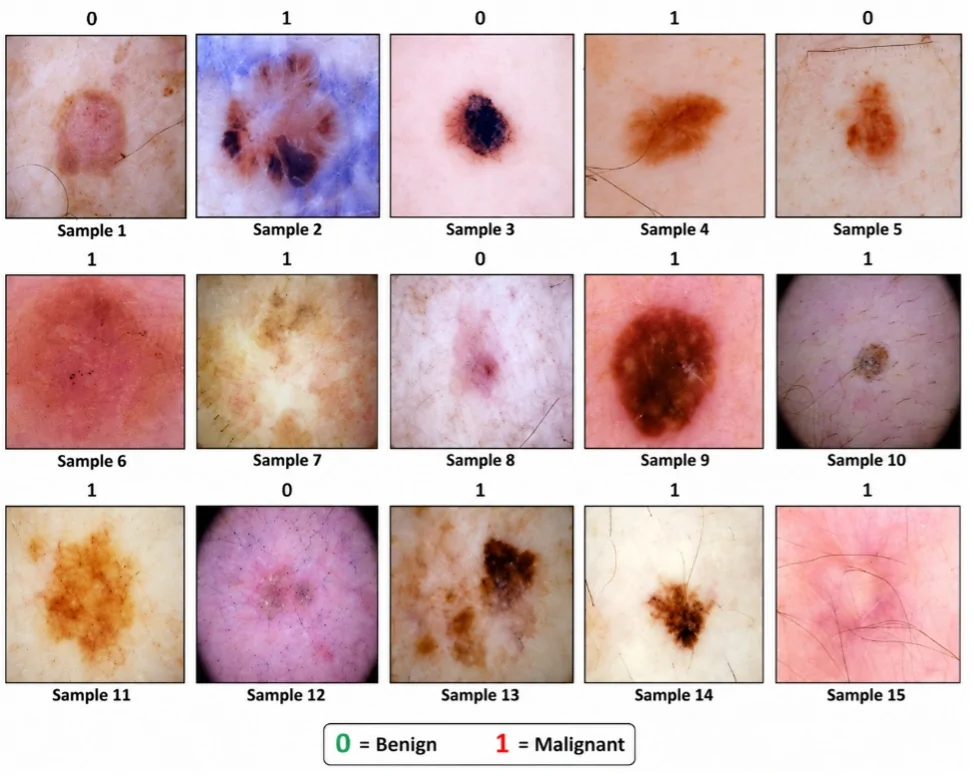
Images from the datasets.

### Model Architecture

#### CNN

The CNN architecture is shown in Figure 2. In general, CNNs are composed of convolution layers, pooling layers, and fully connected layers. The workflow involves input, feature extraction, and classification. Feature extraction is performed using convolutional layers with rectified linear unit (ReLu) activation and pooling layers. Classification is performed using a fully connected layer with softmax activation. The first convolution layer processes the input image by convoluting it through a filter that extracts image features, thereby creating a feature map. Notably, convolution layers process only the data within their receptive fields before forwarding the combined inputs to the subsequent layers. CNN inputs are represented as tensors with specific dimensions; once these tensors pass through the convolutional layer, they are transformed to a feature map. ReLU activation helps reduce errors by improving learning efficiency. Pooling layers help mitigate overfitting by decreasing the spatial dimensions of the feature map [22,23]. Specifically, pooling layers merge the outputs of neuron clusters in one layer into a single neuron in the following layer. The CNN used in this study incorporated average pooling, a technique often used to identify minor changes in images. The fully connected layer lies at the end of the network and connects all neurons of the prior activation layers. Using a flattening process, the resulting maps can be transformed into one-dimensional data [24]. For classification, the flattened matrix is passed to a fully connected layer.

**Figure 2.**
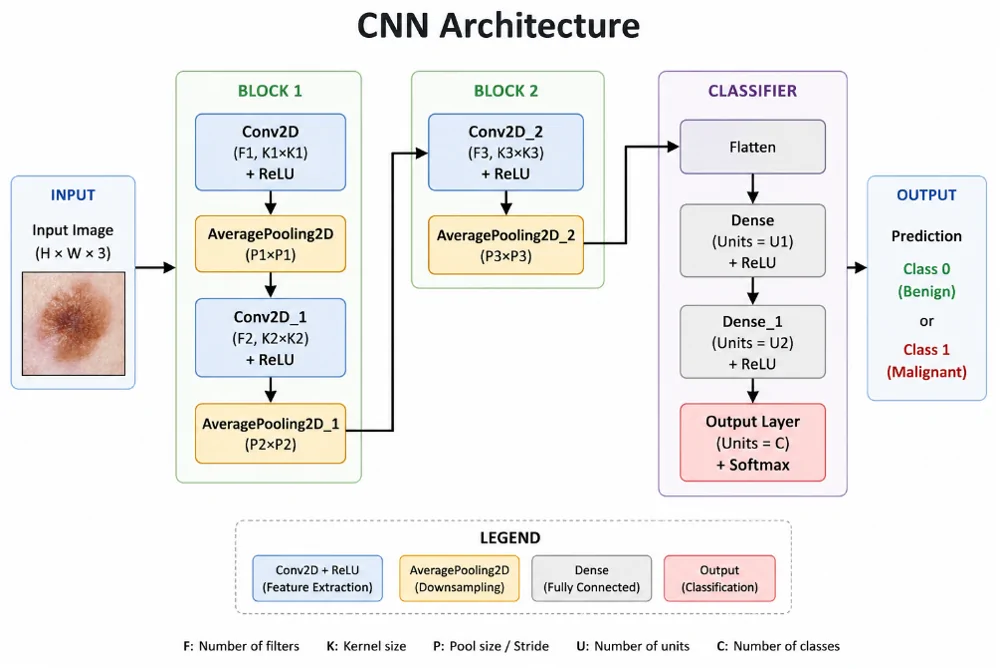
Architecture of the convolutional neural network. CNN: convolutional neural network; ReLU: rectified linear unit.

#### DenseNet

The general architecture of DenseNet is shown in Figure 3. DenseNets are a class of CNNs featuring dense connections between layers with similar feature-map sizes. Specifically, a feedforward connection exists between a given layer and the remaining layers constituting the network. Unlike other convolutional networks that feature a connection only between a given layer and its following layer, DenseNets connect each layer to its previous and following layers [25]. While a standard convolutional network with L layers typically has L connections, a DenseNet with L layers has L(L + 1)/2 direct connections. Each layer in a dense block receives feature maps from all preceding layers and provides its output to all subsequent layers.

**Figure 3.**
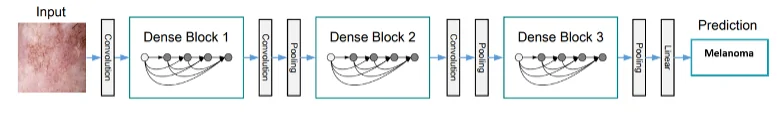
General architecture of DenseNet.

In DenseNets, feature maps obtained from the previous layers are combined through concatenation rather than summing. This architecture offers several advantages, including mitigation of the vanishing gradient problem commonly encountered in CNN frameworks, enhanced strength of connections, more efficient propagation of information, feature reuse, and reduced parameter requirements [26]. The ability of DenseNet to efficiently propagate information facilitates training through implicit deep supervision [27]. DenseNet169 and DenseNet201 include 4 blocks with 6, 12, 32, and 32 layers and 6, 12, 48, and 32 layers, respectively.

#### Proposed Framework

Figure 4 illustrates the framework used to detect skin lesions in this work.

**Figure 4.**
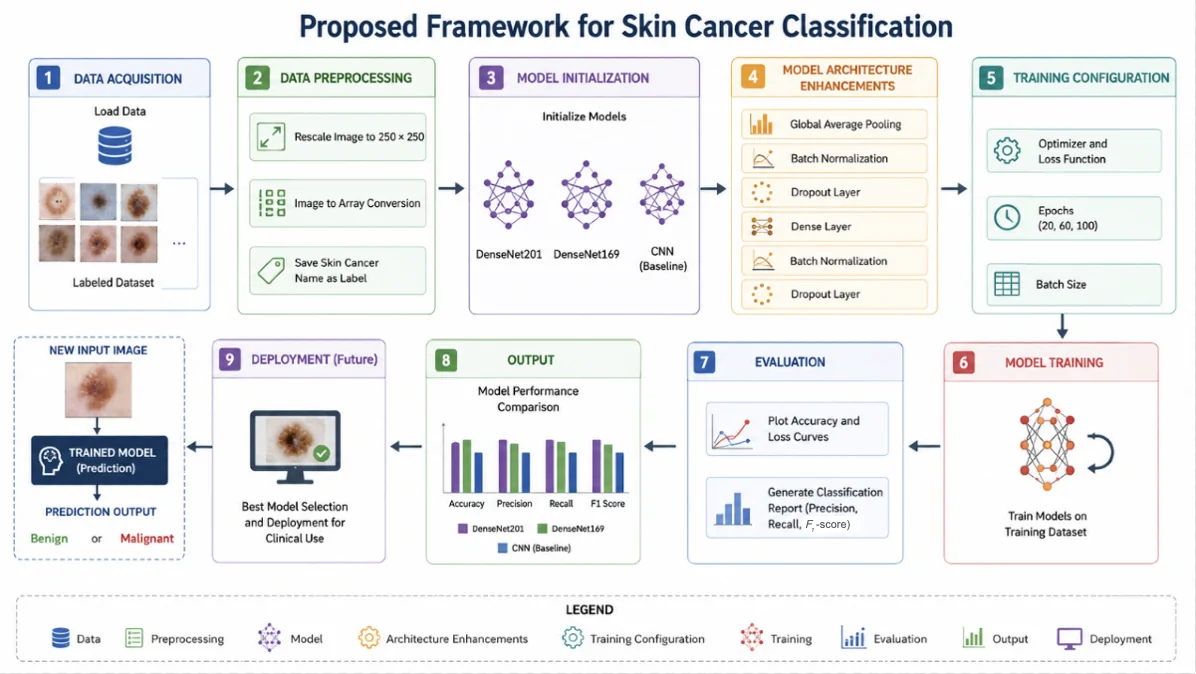
Flowchart of the proposed framework for skin lesion classification. CNN: convolutional neural network.

### Ethical Considerations

This study involved secondary analysis of a publicly available dermoscopic image dataset obtained from Kaggle. No participants were prospectively recruited, and no new patient data or biological specimens were collected. The investigators did not have access to direct patient identifiers or identifiable private information in the dataset used for the analysis. The data were analyzed solely for research purposes in accordance with the terms and conditions of the data source. Because the study was based exclusively on publicly available, deidentified secondary data and involved no direct interaction with or intervention in human participants, informed consent was not obtained for this study. No compensation was provided because no participants were recruited or directly involved.

## Results

### Overview

The models were trained for 20, 60, and 100 epochs. Loss was calculated by subtracting the predicted class values from the actual class values.

The CNN model was first evaluated using the Adam optimizer. At 20 and 60 epochs, training and validation loss decreased gradually, with negligible differences between the 2 values. This indicates that the model did not exhibit overfitting or underfitting at these durations. However, at 100 epochs, training and validation loss exhibited different trends. The validation loss initially decreased but began to increase at 40 epochs. In contrast, the training loss continued to decrease even after 40 epochs. The difference between training and validation loss at 100 epochs indicated overfitting. Similarly, during training for 100 epochs, the training accuracy continued to increase, whereas validation accuracy remained constant at 50%. The *F*_1_-scores on the validation set were 40%, 50%, and 60% at 20, 60, and 100 epochs, respectively. The *F*_1_-score over the complete dataset increased with training duration from 20 to 100 epochs.

The optimizers assessed in this work were RMSprop and Adam. These optimizers affect model performance by influencing the training process. Both optimizers use moving averages of gradients. Although Adam is similar to RMSprop, it integrates momentum-based quantities. Adam has been reported to be more efficient than other optimizers owing to its reduced memory and time consumption, as well as more effective management of scattered gradients in scenarios involving noise [24].

Experiments were conducted using 3 neural network architectures: a conventional CNN, DenseNet169, and DenseNet201. The models were assessed using 2 optimizers (Adam and RMSprop) and over two dataset versions: the original dataset containing 2357 images (version 1) and an augmented dataset containing 6857 images (version 2). Models were trained for 15 to 100 epochs using GPU acceleration. The runtime per epoch ranged from approximately 33 to 64 seconds.

### Model Performance Without Optimizers

#### DenseNet169

The model was trained for 50 epochs. The first epoch took 59 seconds, while the remaining epochs took 33‐39 seconds with GPU acceleration. At the 50th epoch, the model demonstrated strong performance, with a training accuracy of 95.07% and validation accuracy of 94.60%, along with low loss values (training loss of 0.2014 and validation loss of 0.2101), indicating stable learning. The model achieved a high binary accuracy of 0.9616, along with perfect recall (1.0) and strong precision (0.962). These results highlight that the model identified all true positives with few false positives. Figure 5A shows the training and validation accuracy and loss by epoch for DenseNet169.

**Figure 5.**
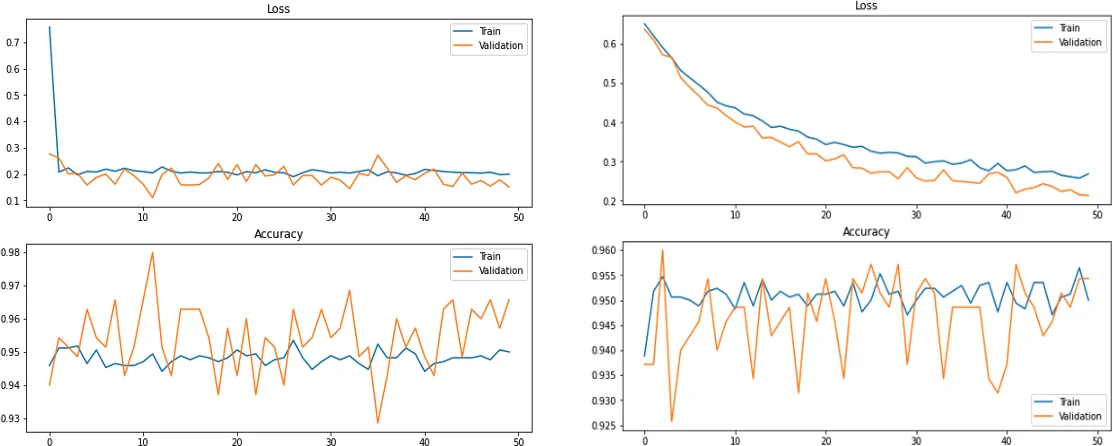
Training and validation accuracy and loss by epoch: (A) DenseNet169, (B) DenseNet201.

#### DenseNet201

The model was trained for 50 epochs. The first epoch took 64 seconds, while the subsequent epochs took 35 seconds with GPU acceleration. At the 50th epoch, the model performed well, achieving a training accuracy of 95% and validation accuracy of 95.43% with low loss values, indicating stable learning. Additionally, the model achieved a binary accuracy of 94.86%, with perfect recall (1.0) and high precision (0.953), indicating it correctly identified all true positives with a low rate of false positives. Figure 5B shows the training and validation accuracy and loss by epoch for DenseNet201.

### Model Performance With Optimizers

The CNN model showed improvements across epochs and optimizers, as discussed below. Adam is an optimization algorithm that can be used as an alternative to classical stochastic gradient descent, iteratively updating network weights based on training data. With the Adam optimizer, validation accuracy on the augmented dataset increased with the training duration, reaching the maximum value of approximately 85% after 100 epochs. RMSprop yielded comparable accuracy but exhibited fluctuations in loss and validation accuracy at higher epochs, suggesting overfitting.

#### Comparison of Results Using RMSprop and Adam

Figure 6 shows the training and validation loss and accuracy of the model at 20, 60, and 100 epochs using the Adam optimizer. At 20 and 60 epochs, training and validation loss gradually decreased, with minimal difference between them. These results indicate that the model did not exhibit overfitting or underfitting. However, at 100 epochs, the validation loss initially decreased but began to increase at approximately 40 epochs. In contrast, training loss continued to decrease after 40 epochs. A similar trend was observed in training and validation accuracy. Training accuracy continuously increased with increasing epoch count, but validation accuracy remained approximately 50%. This deviation indicates overfitting. Figure S1 in Multimedia Appendix 1 shows the confusion matrix for the Adam optimizer.

**Figure 6.**
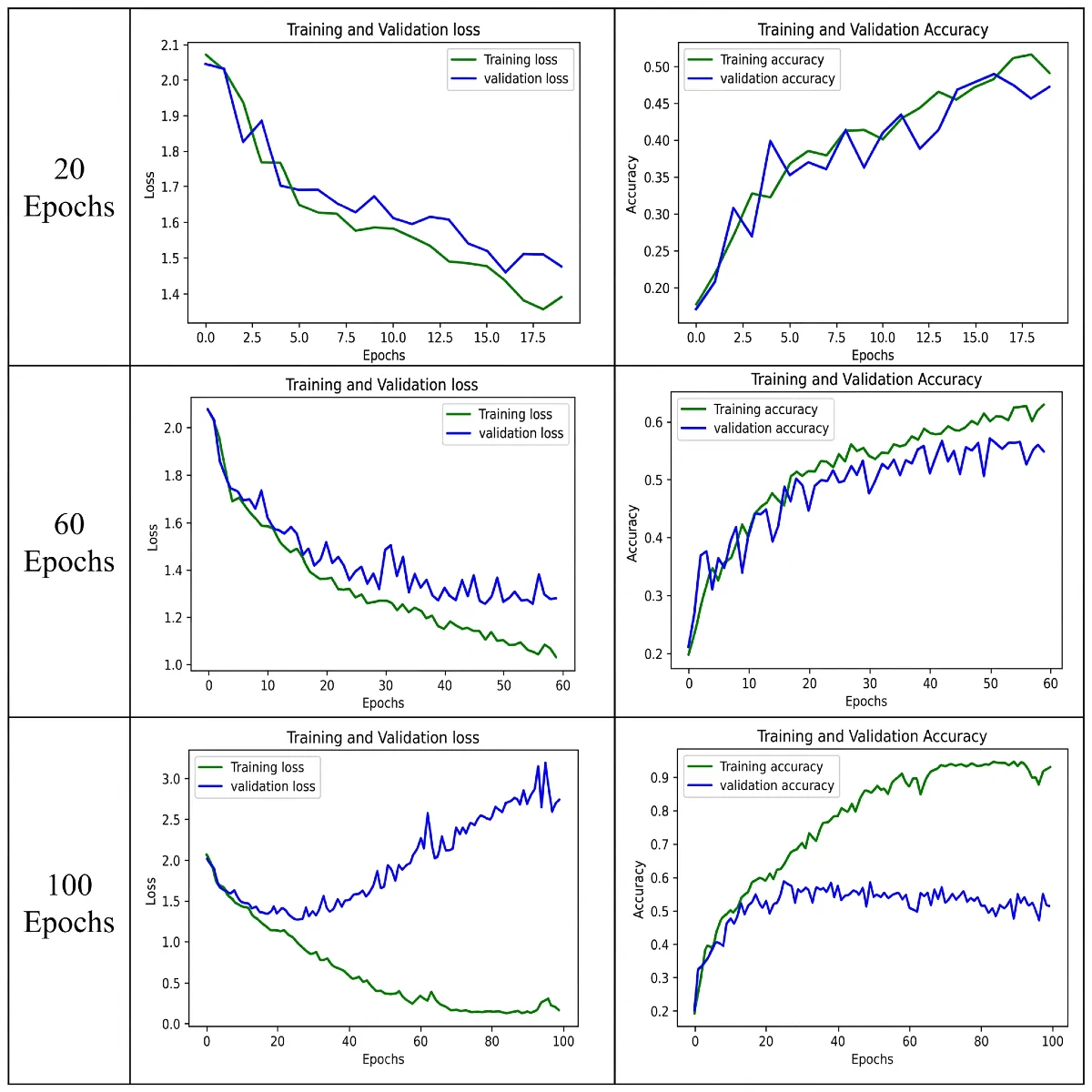
Training and Validation Loss and Accuracy curves for the Adam optimizer. Adam: adaptive moment estimation.

The RMSprop optimizer can be considered similar to the gradient descent algorithm with momentum. This optimizer restricts oscillations in the vertical direction. Therefore, by increasing the learning rate, the model can take larger steps in the horizontal direction, resulting in accelerated convergence. Figure 7 shows the training and validation loss and accuracy of the model at 20, 60, and 100 epochs using the RMSprop Optimizer.

**Figure 7.**
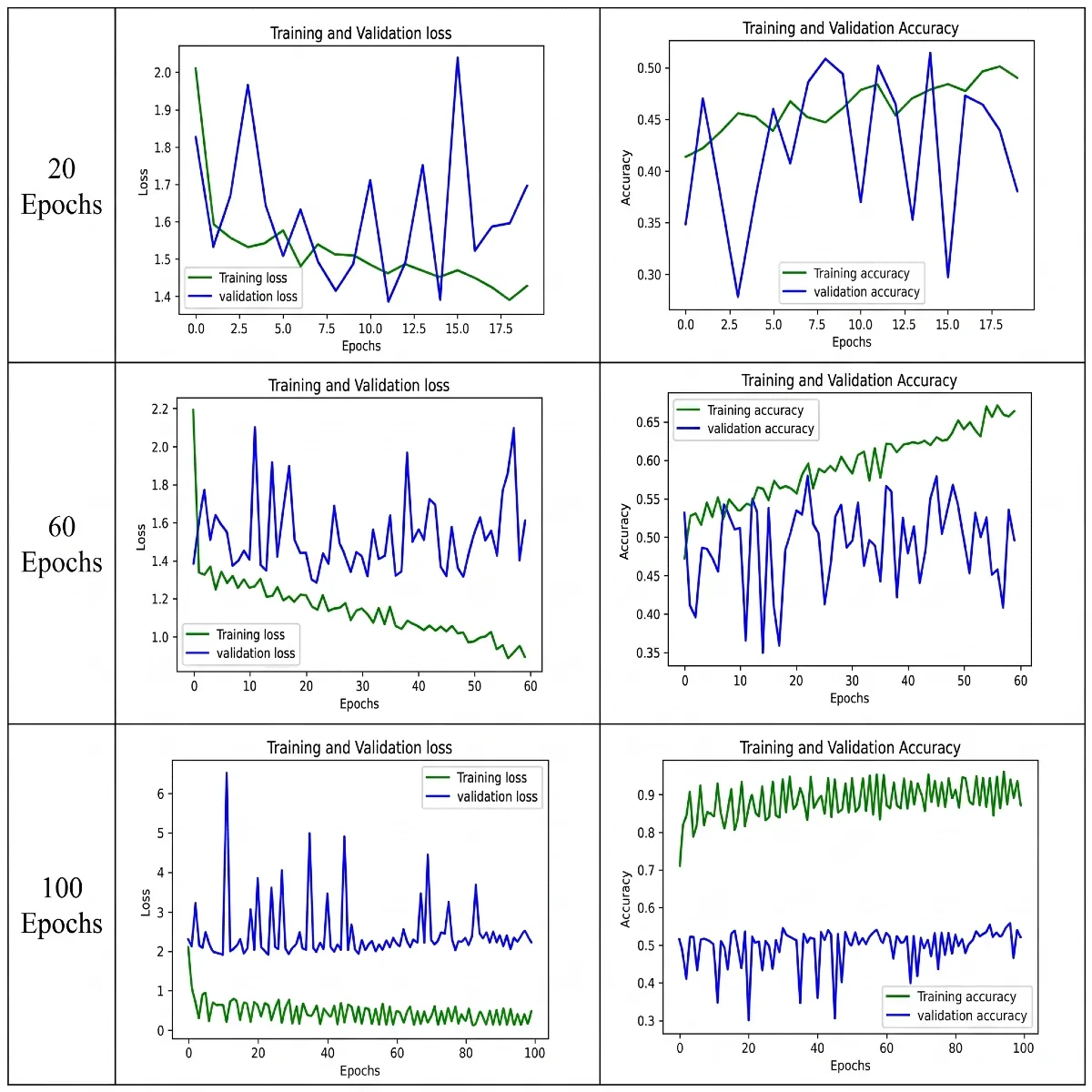
Training and validation loss and accuracy curves for the RMSprop optimizer. RMSprop: root mean square propagation.

The training and validation loss and accuracy curves indicate limited learning efficiency of the model, with sharp fluctuations in both loss and accuracy suggesting overfitting. Figure S2 in Multimedia Appendix 1 shows the confusion matrix for the RMSprop optimizer.

For the complete dataset, the *F*_1_-scores were 31%, 59%, and 87% at 20, 60, and 100 epochs, respectively. Even though the *F*_1_-score was 87% after 100 epochs, the model performance was expected to be unsatisfactory on unseen data (data not in the training and validation sets).

The results indicate that the model overfit after 100 epochs. Lack of sufficient data may contribute to overfitting. Both the Adam and RMSprop optimizers displayed similar *F*_1_-scores after 100 epochs. Although both optimizers exhibit momentum-like behavior (Adam through its bias-corrected moving average and RMSprop with momentum by explicitly adding a fraction of the previous step), the mechanisms are different, which may influence the hyperparameter learning rate.

To enhance model performance, data augmentation was performed using shear rotations of ±10°. Although this transformation led to only minor changes in the existing data, the model treated them as newly input images. Approximately 500 images were added per class to ensure a balanced class distribution.

The CNN was then trained using the augmented dataset. Figures 8 and 9 show the loss and accuracy associated with the augmented training and validation data at 20, 60, and 100 epochs using the Adam and RMSprop optimizers, respectively. Figures S3 and S4 in Multimedia Appendix 1 present the confusion matrices for the augmented validation data using the Adam and RMSprop optimizers, respectively.

**Figure 8.**
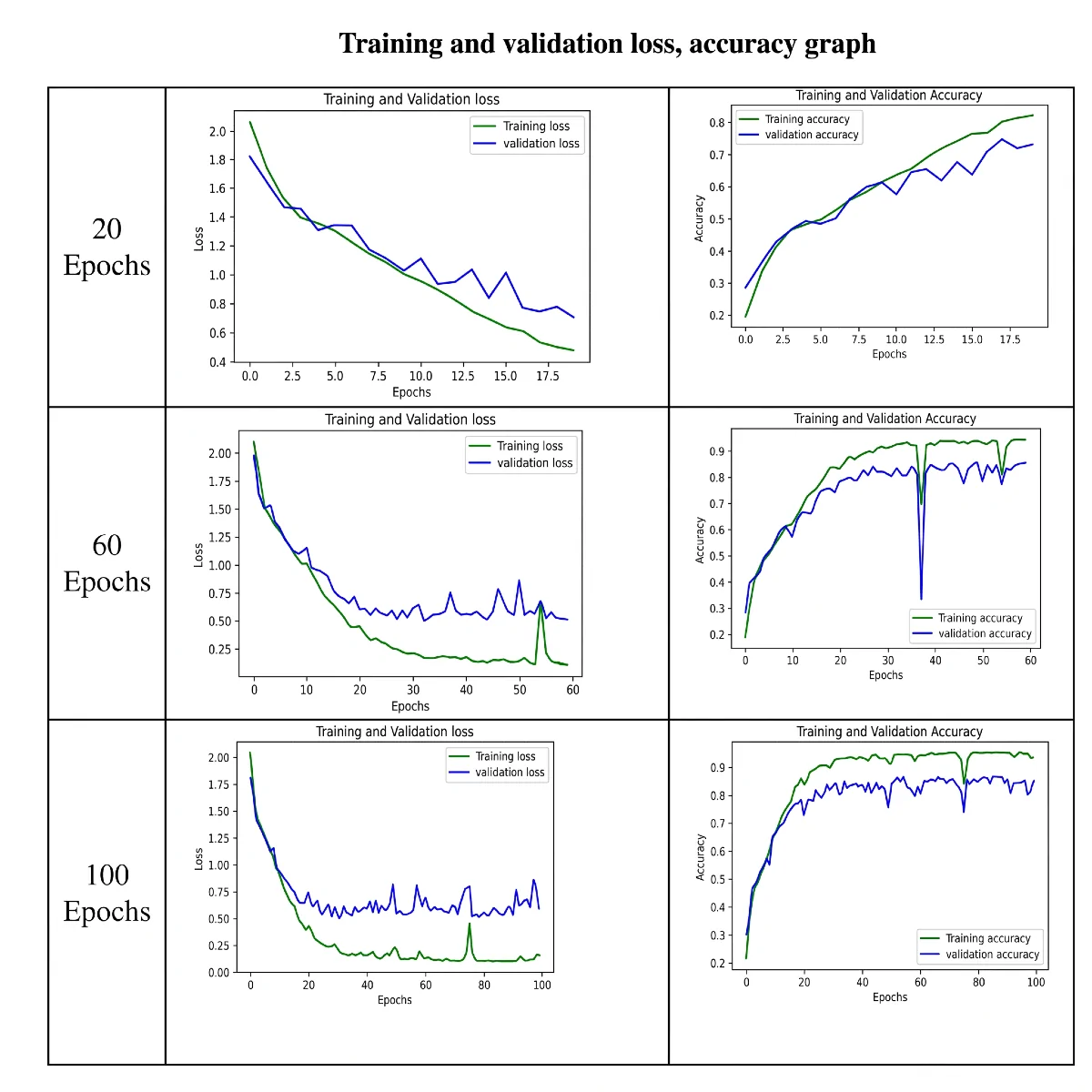
Training and validation loss and accuracy curves for the augmented dataset using the Adam optimizer at 20, 60, and 100 epochs. Adam: adaptive moment estimation.

**Figure 9.**
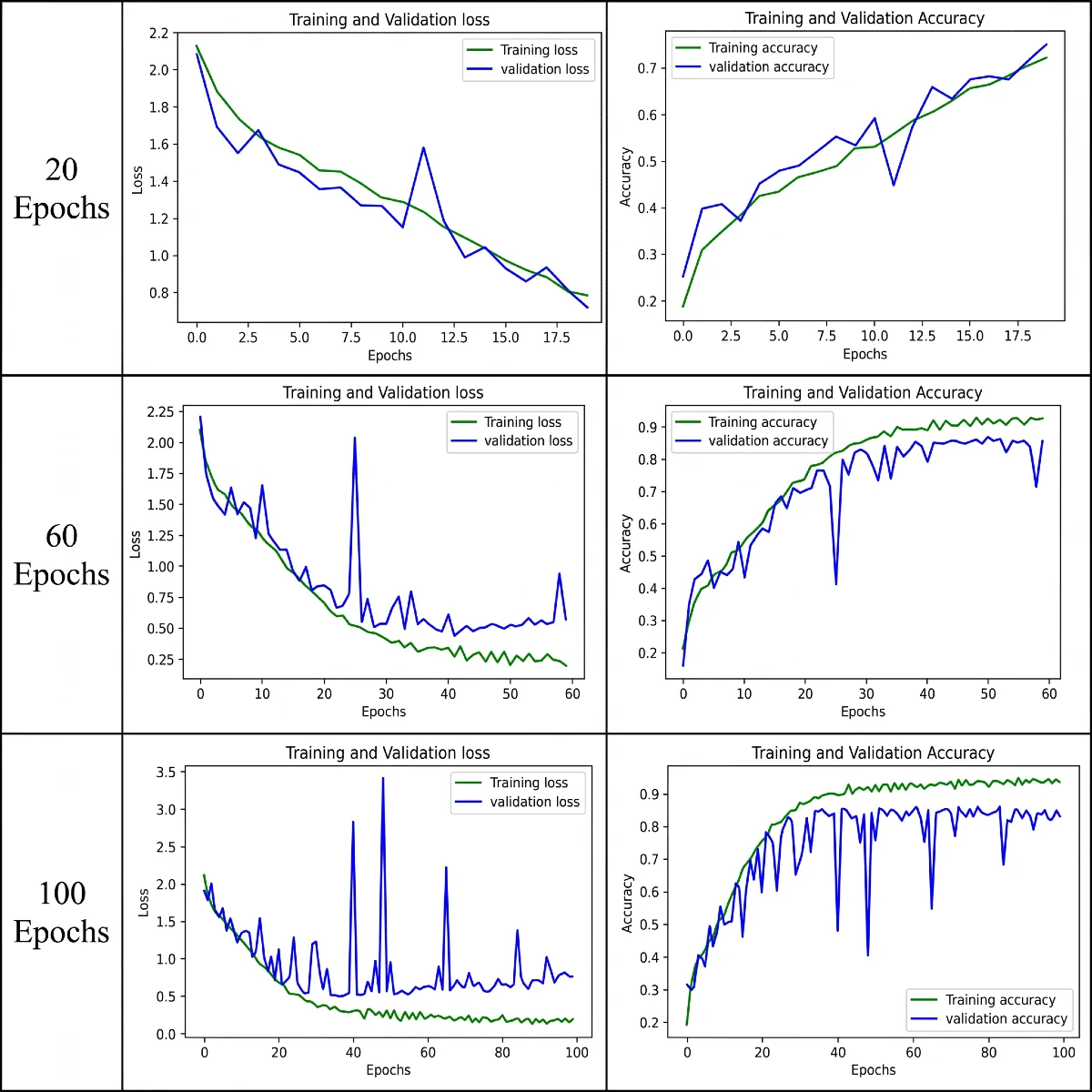
Training and validation loss and accuracy curves for the augmented dataset using the RMSprop optimizer at 20, 60, and 100 epochs. RMSprop: root mean square propagation.

As shown in Figure 8, training and validation loss decreased compared with the results obtained using the original dataset. At 100 epochs, the training and validation accuracy exceeded 90% and 85%, respectively. Although both loss and accuracy improved with the RMSprop optimizer as well (Figure 9), sudden increases in loss and corresponding decreases in accuracy were noted.

#### DenseNet169

Figures 10 and 11 and Figures S5 and S6 in Multimedia Appendix 1 summarize the performance of DenseNet169 in terms of accuracy, stability, and generalizability. Figures 10 and 11 present the training and validation loss and accuracy curves using the Adam and RMSprop optimizers, respectively. Figures S5 and S6 in Multimedia Appendix 1 present the confusion matrices for the validation set using the Adam and RMSprop optimizers, respectively.

**Figure 10.**
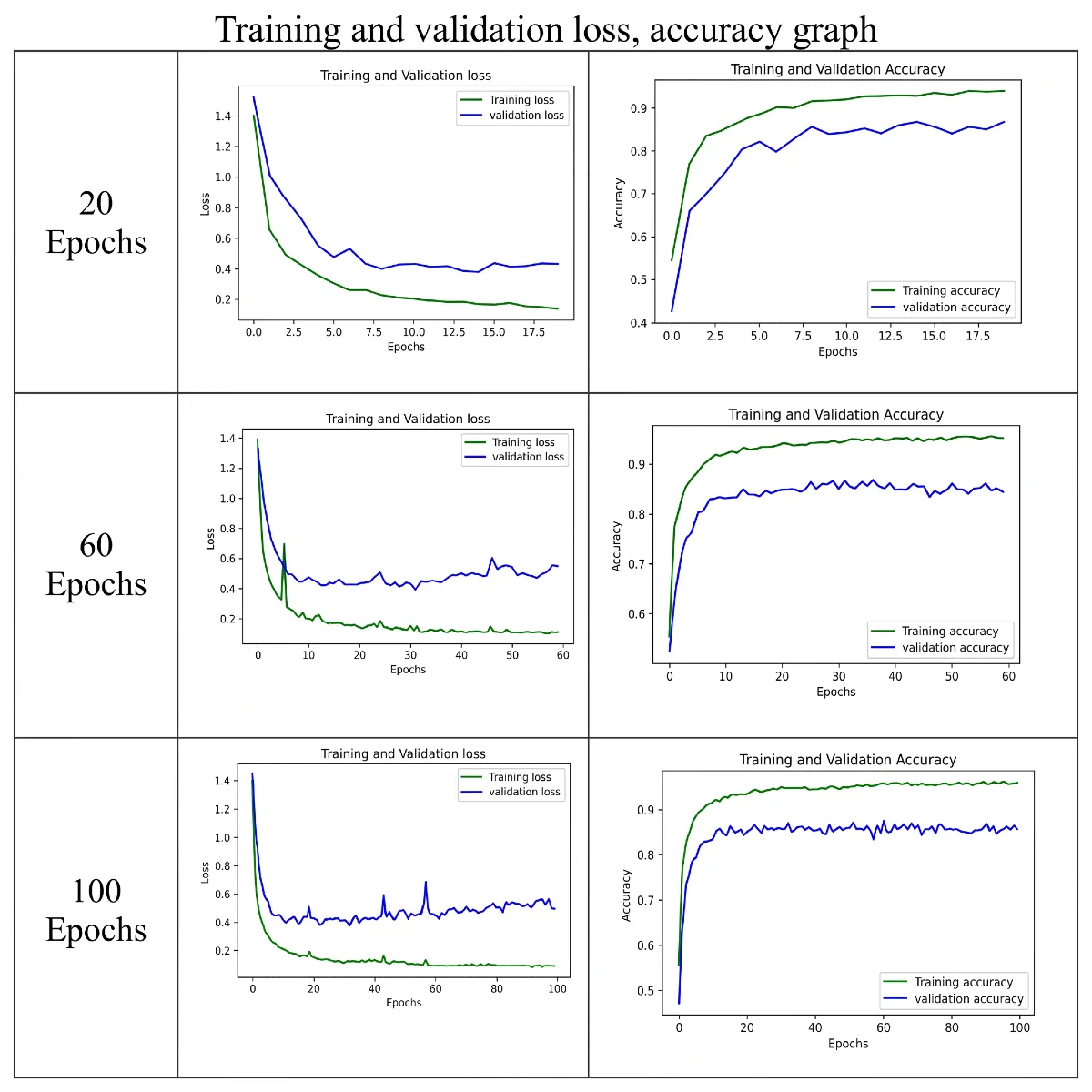
Training and validation loss and accuracy curves for DenseNet169 using the Adam optimizer at 20, 60, and 100 epochs. Adam: adaptive moment estimation.

**Figure 11.**
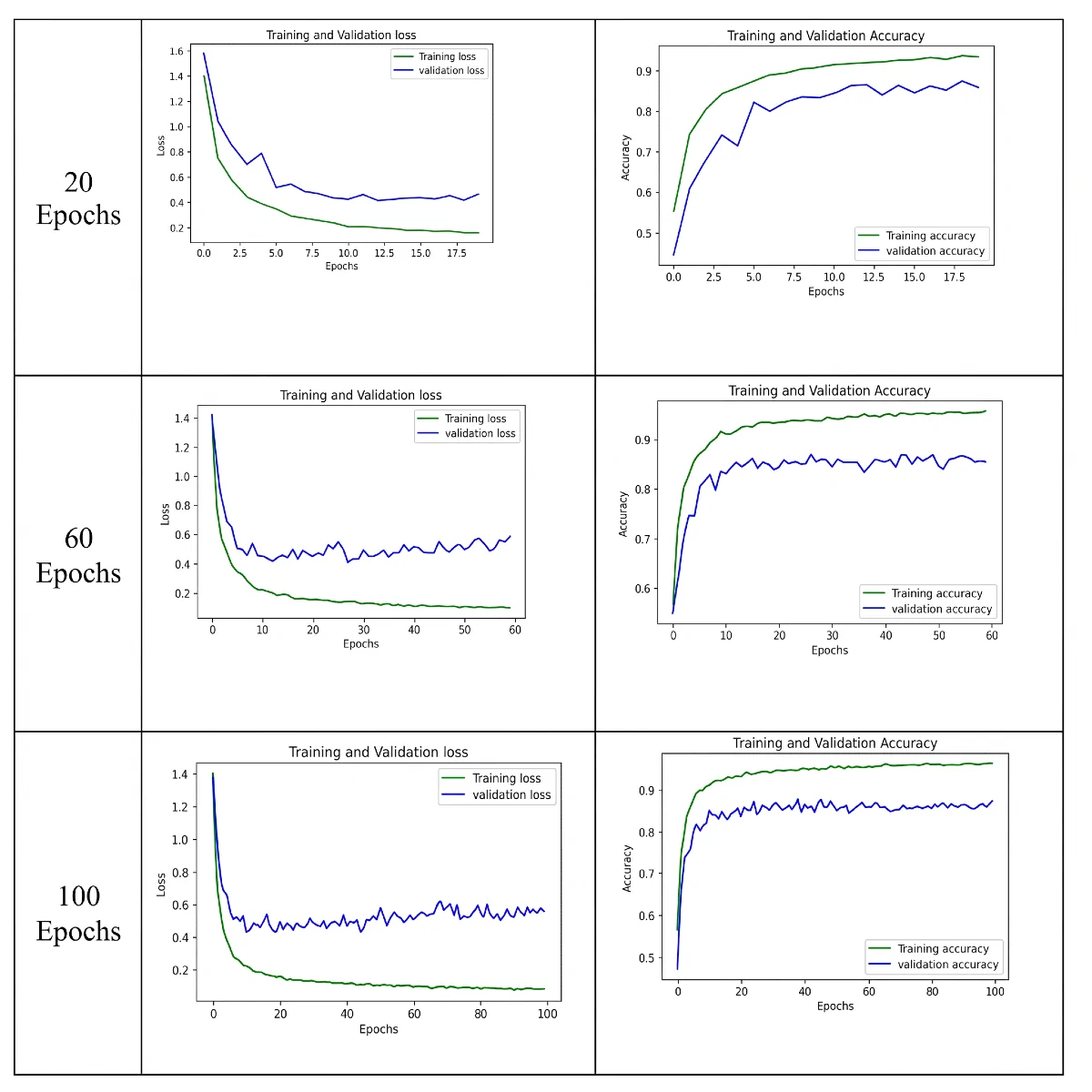
Training and validation loss and accuracy curves for DenseNet169 using the RMSprop optimizer at 20, 60, and 100 epochs. RMSprop: root mean square propagation.

With increase in the dataset size, DenseNet169 achieved higher accuracy and smaller loss than those for the CNN architecture at 20, 40, and 60 epochs. Validation accuracy was 85%

#### DenseNet201

Figures 12 and 13 and Figures S7 and S8 in Multimedia Appendix 1 summarize the performance of DenseNet201 in terms of accuracy, stability, and generalizability across the two optimization strategies. Figures 12 and 13 present the training and validation loss and accuracy curves using the Adam and RMSprop optimizers, respectively. Figures S7 and S8 in Multimedia Appendix 1 present the confusion matrices for the validation set using the Adam and RMSprop optimizers, respectively.

**Figure 12.**
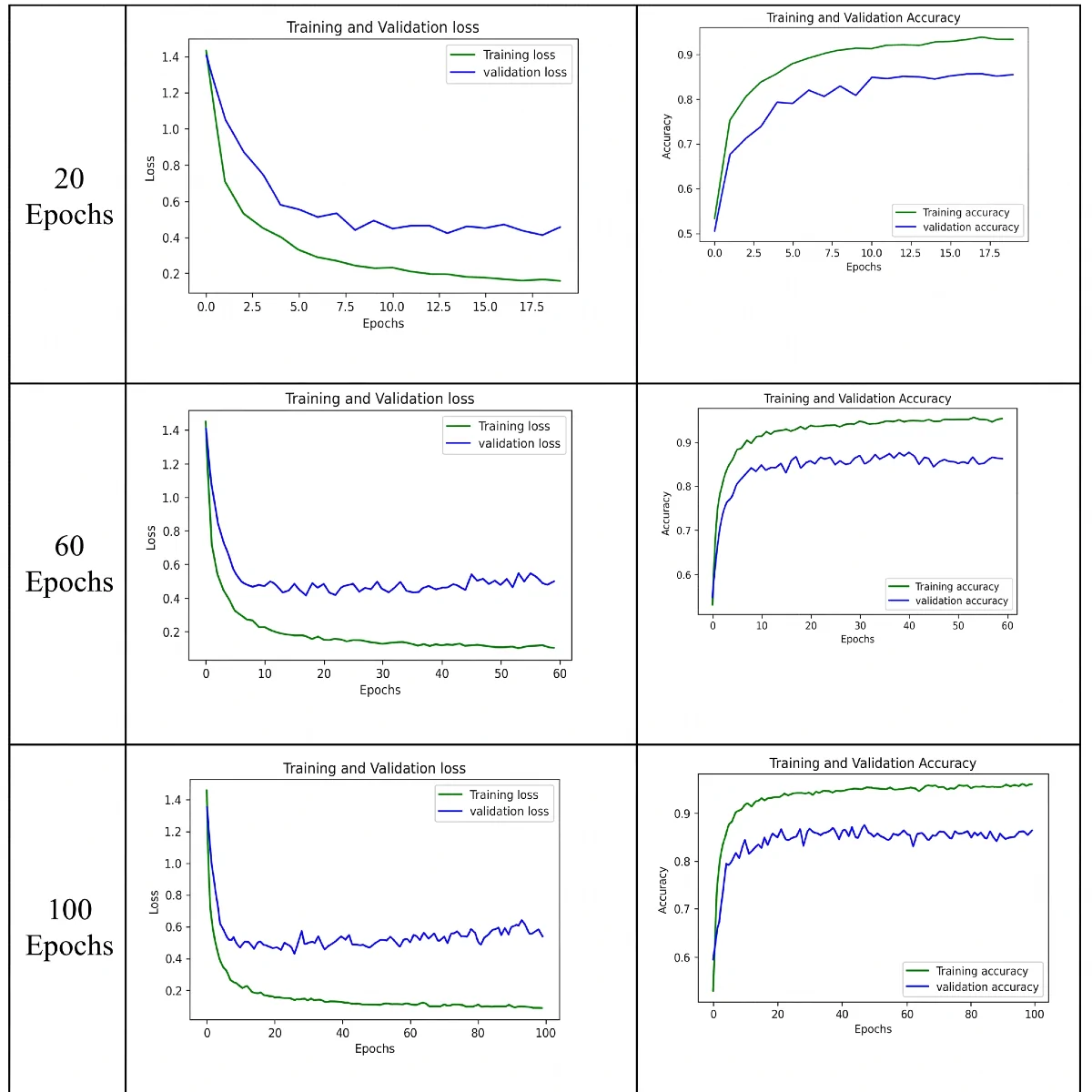
Training and validation loss and accuracy curves for DenseNet201 using the Adam optimizer at 20, 60, and 100 epochs. Adam: adaptive moment estimation.

**Figure 13.**
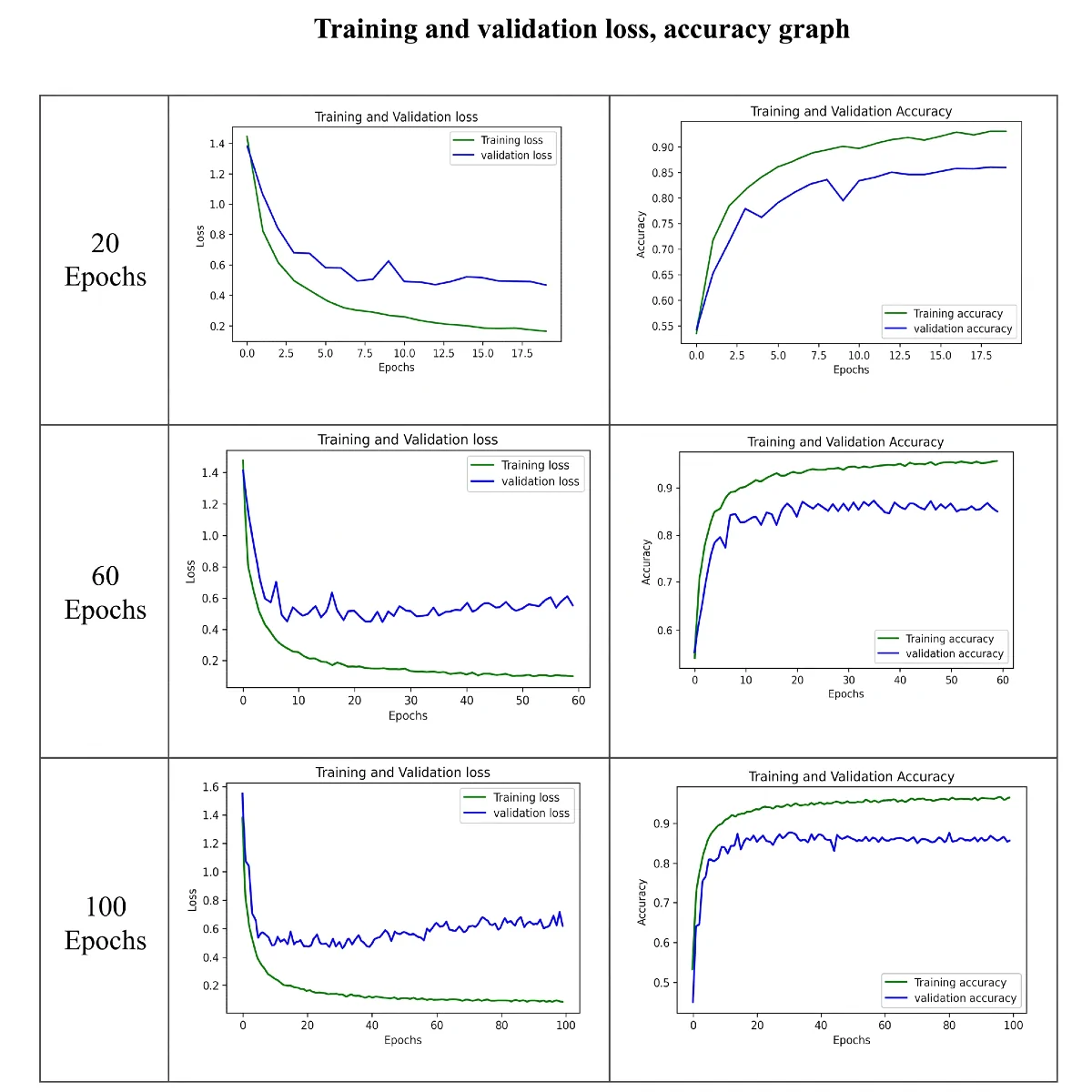
Training and validation loss and accuracy curves for DenseNet201 using the RMSprop optimizer at 20, 60, and 100 epochs. RMSprop: root mean square propagation.

#### Comparative Analysis

Both DenseNet variants consistently outperformed the CNN baseline. On the augmented data, both models achieved accuracy and *F*_1_-scores exceeding 94% after 100 epochs, with only minor differences between the Adam and RMSprop optimizers. DenseNet169 achieved a validation accuracy of up to 95% and demonstrated superior generalization benefits compared with both CNN and DenseNet201, attributable to its dense connectivity pattern.

Augmentation involved generating approximately 500 synthetic samples per class using image transformations such as ±10° shear, resulting in improved class balance and model generalizability. The augmented dataset (version 2) was associated with increased accuracy and more stable validation loss curves, suggesting potential reduction in overfitting. Figure 14 shows heatmaps of skin lesion images from the dataset.

**Figure 14.**
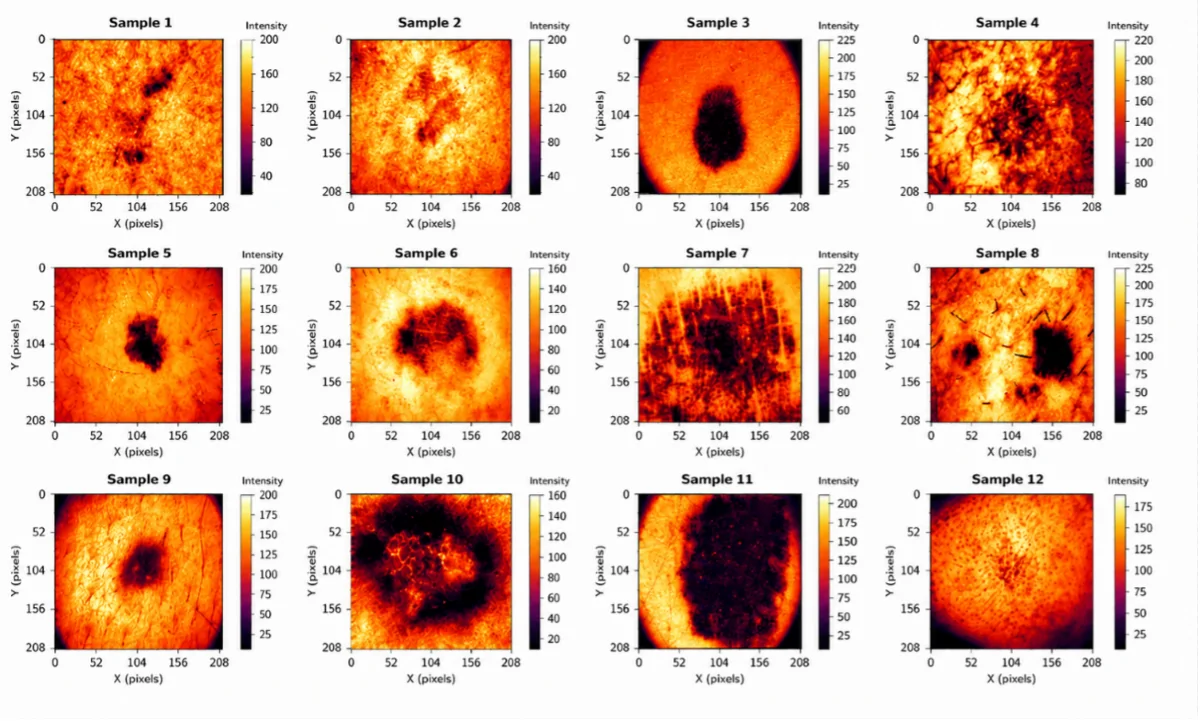
Heatmaps of skin lesion images from the dataset.

Table 2 presents the accuracy values of the CNN, DenseNet169, and DenseNet201 models. Data were obtained from Kaggle, and the models were also tested on an augmented dataset containing additional samples for each class. The dataset was divided using an 80:20 split. The validation set corresponded to the 20% portion, and the “whole dataset” included both the training and validation sets (80%+20%).

**Table 2.** Overall performance (accuracy, %) of the algorithms.

Model and optimizer	Dataset (2357 samples)						Dataset (6857 samples)					
	Validation data			Whole dataset			Validation data			Whole dataset		
	20 epochs	60 epochs	100 epochs	20 epochs	60 epochs	100 epochs	20 epochs	60 epochs	100 epochs	20 epochs	60 epochs	100 epochs
CNN^a^												
Adam^b^	42	52	50	44	59	86	72	86	85	82	94	93
RMSprop^c^	31	48	52	31	59	87	76	86	83	82	94	93
DenseNet169												
Adam	58	58	58	87	88	88	87	84	86	94	94	95
RMSprop	55	57	60	85	87	88	86	86	87	94	95	95
DenseNet201												
Adam	56	58	57	87	88	88	85	86	86	94	95	95
RMSprop	56	56	58	86	88	88	86	84	85	93	94	95

a CNN: convolutional neural network.

b Adam: adaptive moment estimation.

c RMSprop: root mean square propagation.

Figure 15 compares the accuracy of the three studied models, highlighting the superior accuracy of DenseNet169 and DenseNet201 compared with the conventional CNN architecture.

**Figure 15.**
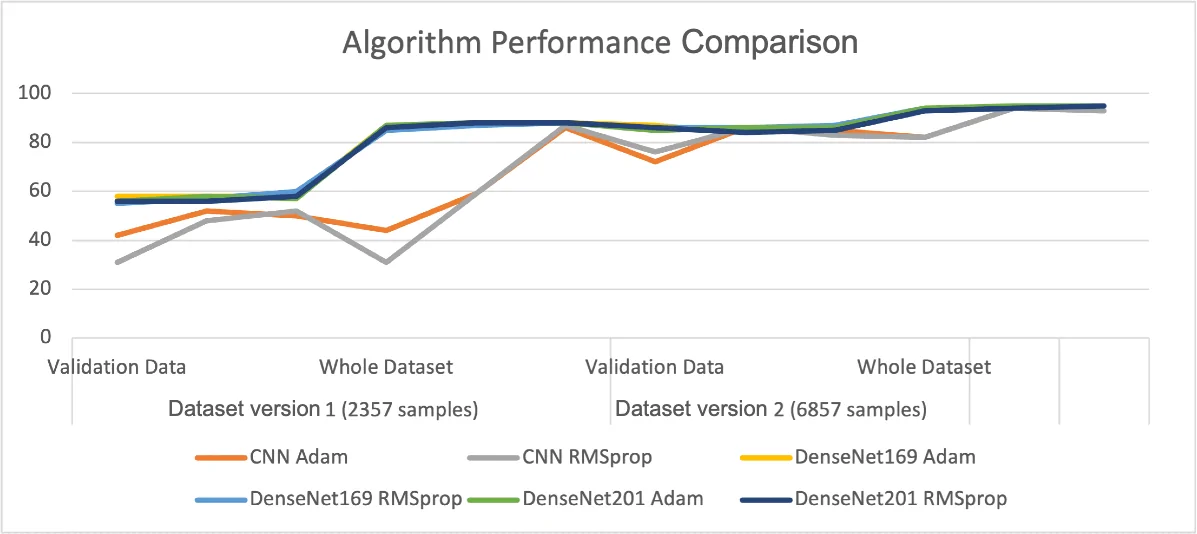
Algorithm performance comparison. Adam: adaptive moment estimation; CNN: convolutional neural network; RMSprop: root mean square propagation.

## Discussion

### Implications

AI is increasingly transforming medicine by enhancing diagnostic accuracy, enabling personalized treatment strategies, and improving overall patient outcomes. Its ability to rapidly process large datasets, identify complex patterns, and support clinical decision-making is reshaping health care delivery. Notably, AI systems can improve diagnostic reliability, reduce human error, and streamline clinical workflows across medical specialties.

In dermatology, AI has garnered considerable attention for its potential to improve the early detection of skin cancer, particularly melanoma. Advanced deep learning models, including CNNs and DenseNet architectures, have demonstrated performance comparable to, and in some cases, exceeding that of dermatologists in the analysis of dermoscopic and clinical images. These approaches can help address inherent challenges, such as subjective interpretation and interobserver variability, offering scalable and consistent solutions for large-scale skin cancer screening.

The integration of CNN and DenseNet architecture into a unified diagnostic framework is especially valuable in clinical practice. When embedded within dermatology workflows as a decision support tool, such systems can aid physicians by automating lesion analysis. Such automation could help reduce diagnostic uncertainty, enhance clinician confidence, and facilitate the efficient classification of multiple lesion types, enabling prioritization of high-risk cases and timely decision-making.

The proposed automated framework incorporates advanced image preprocessing and augmentation techniques to improve model robustness and generalizability. By minimizing reliance on subjective visual assessment and complementing traditional dermoscopy, the system can support earlier and more consistent melanoma detection. Dense connectivity facilitates the extraction of subtle yet clinically relevant features, improving the discrimination of benign and malignant lesions.

AI-assisted analysis can also contribute to improved clinical efficiency. Notably, rapid and accurate lesion classification can accelerate diagnostic workflows, reduce the time spent on image interpretation, and shorten patient visits, potentially yielding an increase in patient throughput while maintaining diagnostic quality. Additionally, AI-driven triage may allow trained technical staff to assist with image acquisition and preliminary screening, optimizing resource utilization within clinical settings.

From an economic perspective, AI implementation in dermatology may have the potential to reduce health care costs. Early and accurate detection of melanoma could decrease unnecessary biopsies and limit the need for costly treatments associated with advanced-stage disease. By identifying high-risk lesions earlier, the proposed framework could support more effective resource allocation, ultimately improving both economic aspects and patient outcomes.

Moreover, automation of routine image analysis may allow clinical staff to devote more time to patient coordination and care delivery. This redistribution of responsibilities would enhance operational efficiency and may help reduce provider burnout.

The compatibility of the proposed framework with dermoscopic imaging may support the use of AI in teledermatology. Patients could remotely submit lesion images for preliminary assessment, enabling prioritization of urgent cases and enhancing access to care in underserved or remote regions. This capability could strengthen continuity of care through remote monitoring and timely intervention.

Future work could be focused on improving model interpretability and performance through the integration of attention mechanisms into DenseNet architectures. These mechanisms could allow the model to highlight salient image regions, enhancing both diagnostic accuracy and transparency, which may increase clinician trust. Additionally, incorporating multimodal data—such as patient demographics and clinical history—alongside imaging features may further improve diagnostic precision. Validation across diverse populations and real-world clinical settings will be essential to ensure generalizability and support widespread clinical adoption.

Although the findings of this study are encouraging and suggest the potential clinical utility of the proposed framework, further validation is necessary before routine clinical implementation. Specifically, prospective evaluation across multiple institutions using larger and independent patient cohorts will be essential to assess generalizability across patient populations, imaging systems, clinical environments, and real-world workflows. Such multicenter validation may also help identify potential sources of variability and bias that may not be fully captured within the current dataset. Future studies should therefore prioritize prospective external validation to establish the robustness, reproducibility, and clinical reliability of the framework before its integration into routine clinical practice.

### Clinical Translation and Future Deployment

Although the proposed CNN and DenseNet architectures demonstrated strong diagnostic performance on the evaluated dataset, clinical deployment requires validation beyond retrospective benchmarking. Before implementation in routine dermatology practice, the models should undergo prospective, multicenter external validation using images acquired from diverse populations, imaging devices, and clinical environments to assess generalizability. Integration into clinical workflows will also require consideration of computational efficiency, interoperability with electronic health record systems, regulatory approval, data privacy, and clinician acceptance. Continuous performance monitoring and periodic model retraining will be essential to mitigate performance degradation resulting from distribution shifts in real-world data. Future work should therefore focus on external validation, explainability, uncertainty estimation, and workflow integration to facilitate the safe and effective adoption of these models as clinical decision support systems.

### Conclusion

This study presented a comprehensive comparative evaluation of CNN and DenseNet-based deep learning architectures for automated classification of melanoma using dermoscopic skin lesion images. In experiments conducted under multiple training configurations, the DenseNet models consistently demonstrated superior classification performance compared with the baseline CNN architecture, with higher accuracy, sensitivity, specificity, precision, and *F*_1_-score. These results highlight that dense feature propagation and feature reuse may benefit the identification of malignant skin lesions while reducing false positive and false negative predictions. The presented comparison of commonly used deep learning architectures can guide researchers and clinicians in selecting appropriate models for computer-aided melanoma diagnosis. The proposed framework can serve as a clinical decision support tool, assisting dermatologists in the early detection of melanoma, thereby facilitating timely intervention and improving patient outcomes. Although further prospective, multicenter external validation is required before routine clinical implementation, the findings demonstrate the potential of deep learning–based image analysis in enhancing diagnostic accuracy and supporting precision dermatology. Overall, this work contributes to the growing body of evidence supporting the integration of AI into dermatological practice and establishes a strong foundation for future research on robust, interpretable, and clinically deployable skin cancer screening systems.

## Supplementary material

10.2196/104086 Multimedia Appendix 1 Supplemental figures S1 to S8, showing confusion matrices for model validation performance using adaptive moment estimation (Adam) and root mean square propagation (RMSprop) optimizers.

## Abbreviations

ABCDE: asymmetry, border irregularity, color heterogeneity, diameter, and evolution
Adam: adaptive moment estimation
CNN: convolutional neural network
DenseNet: densely connected neural network
NMSC: nonmelanoma skin cancer
ReLu: rectified linear unit
RMSprop: root mean square propagation
