# Supplementary material for: Deep Learning–Based Classification of Melanoma Using Dermoscopic Images: Comparative Evaluation of Convolutional Neural Network and DenseNet Models
Source: JMIR Dermatol. 2026 Oct 27;9:e104086. doi: 10.2196/104086 (PMC13717340; doi:10.2196/104086)
Supplement: Supplementary file 1 [file derma-v9-e104086-s001.docx]

Multimedia Appendix 1

*Supplemental Figures*

Figure S1 Confusion matrix on validation data for Adam Optimizer

|   20 Epochs |   60 Epochs |
| --- | --- |
|   100 Epochs | |

Figure S2 Confusion matrix on validation data for RMSprop Optimizer

|   20 Epochs |   60 Epochs |
| --- | --- |
|   100 Epochs | |

Figure S3 Confusion matrix on validation data for Adam Optimizer

|   20 Epochs |   60 Epochs |
| --- | --- |
|   100 Epochs | |

Figure S4 Confusion matrix on validation data on RMS Prop Optimizer

|   20 Epochs |   60 Epochs |
| --- | --- |
|    100 Epochs | |

Figure S5 Confusion matrix on validation data for Adam Optimizer

|   20 Epochs |   60 Epochs |
| --- | --- |
|   100 Epochs | |

Figure S6 Confusion matrix on validation data for RMSprop Optimizer

|   20 Epochs |   60 Epochs |
| --- | --- |
|   100 Epochs | |

*Figure S7 Table: Confusion matrix on validation data for Adam Optimizer*

|   20 Epochs |   60 Epochs |
| --- | --- |
|   100 Epochs | |

Figure S8 Confusion matrix on validation data for RMSprop Optimizer

|   20 Epochs |   60 Epochs |
| --- | --- |
|   100 Epochs | |
